# ‘It gives me purpose’: stories shared by Aboriginal mothers and their perspectives on nurturing resilience

**DOI:** 10.1080/00049530.2025.2555650

**Published:** 2025-09-17

**Authors:** Charlotte Sapio, Natasha J. Howard, Tina Brodie, Karen Glover, Renae Holmberg, Yvonne Clark

**Affiliations:** a Aboriginal Communities and Families Health Research Alliance (ACRA), SAHMRI, Adelaide, South Australia, Australia; b Justice and Society, University of South Australia, Adelaide, South Australia, Australia; c Faculty of Health and Medical Sciences, University of Adelaide, Adelaide, South Australia, Australia; d Wardliparingga Aboriginal Health Equity, SAHMRI, Adelaide, South Australia, Australia

**Keywords:** Aboriginal, motherhood, resilience, social and emotional wellbeing, strength based approach

## Abstract

**Objective:**

Aboriginal and Torres Strait Islander women have unique experiences of motherhood, underpinned by intergenerational cultural knowledge and holistic practices. Ongoing colonial violence perpetuates adversity associated with peri- and post-natal health and wellbeing outcomes. Aboriginal mothers’ perceptions of resilience are not well understood, with resilience predominantly framed by Eurocentric understandings. Subsequently, this research explored the gap of post-natal resilience from an Aboriginal perspective.

**Method:**

The research expands upon the “Corka Bubs” research of Aboriginal mothers in the antenatal period which sought to develop a novel care package to reduce adverse experiences. Utilising an Indigenous methodological lens, yarning took place with five mothers and the transcribed material was thematically analysed.

**Results:**

Four core themes for Aboriginal resilience in motherhood were identified: Connection, Learning and Growing, Caring for Self and Others, and Identity. Combined, these connections enabled mothers to combat adversity and remain strong for their children.

**Conclusion:**

The stories shared contribute to strengths-based understandings of Aboriginal resilience. Our findings suggest that Aboriginal mothers' resilience is grounded within holistic and collectivist values, differing from Western perceptions of resilience.

## KEY POINTS

**What is already known about this topic:**

1. Aboriginal communities’ conceptualisations of health and wellbeing encapsulate connection to holistic and cultural influences, which nurture positive health outcomes.
2. The ongoing oppression of Aboriginal Peoples has resulted in Aboriginal women experiencing preventable adverse pre- and postnatal outcomes.
3. Resilience is reflected as an essential trait or inherent process needed for all humans across the lifespan to adapt and overcome adversity.

**What this topic adds:**

1. Qualitative information regarding the ways Aboriginal women stay strong within a postnatal context.
2. Provides an understanding of the cultural and colonial factors that inform resilience for Aboriginal mothers.
3. Shapes knowledge of a culturally informed and strength-based perspective of Aboriginal Resilience.

## Introduction

### Colonial context of wellbeing

The colonisation and attempted genocide of Australia’s Aboriginal and Torres Strait Islander Peoples continue to impact health and wellbeing outcomes for communities (Adane et al., 2023; Ussher et al., 2016; Weetra et al., 2016). Aboriginal and Torres Strait Islander Peoples have been forced to assimilate to Western ways of living under paternalistic and political agendas to forgo much of their intergenerational cultural practices and knowledge systems. The experiences of loss and grief and intergenerational trauma has lessened the opportunity for Aboriginal and Torres Strait Islander communities^1^ to live healthy and functional lives (Adane et al., 2023; Gibberd et al., 2019; Ussher et al., 2016). Aboriginal women and mothers are the backbone of Aboriginal and Torres Strait Islander communities and are strong survivors yet experience chronic adversity in the maternal role. Specifically, Aboriginal mothers experience higher rates of mental ill-health; preterm birth and perinatal mortality; child protection removals; substance use; chronic disease; adolescent pregnancy; socio-economic disadvantage; racism and discrimination; incarceration; decreased literacy skills; and insufficient access to culturally safe services (Simpson et al., 2020; Sivertsen et al., 2022; Ussher et al., 2016). Whilst Aboriginal mothers experience complex adversities as part of their maternal role, it is unclear how resilience is used as a protective factor. Psychology has a core and obligatory role in addressing the longstanding impacts of intergenerational trauma to support thriving mothers and children.

### Aboriginal ways of being, knowing, and doing

**Figure 1.**
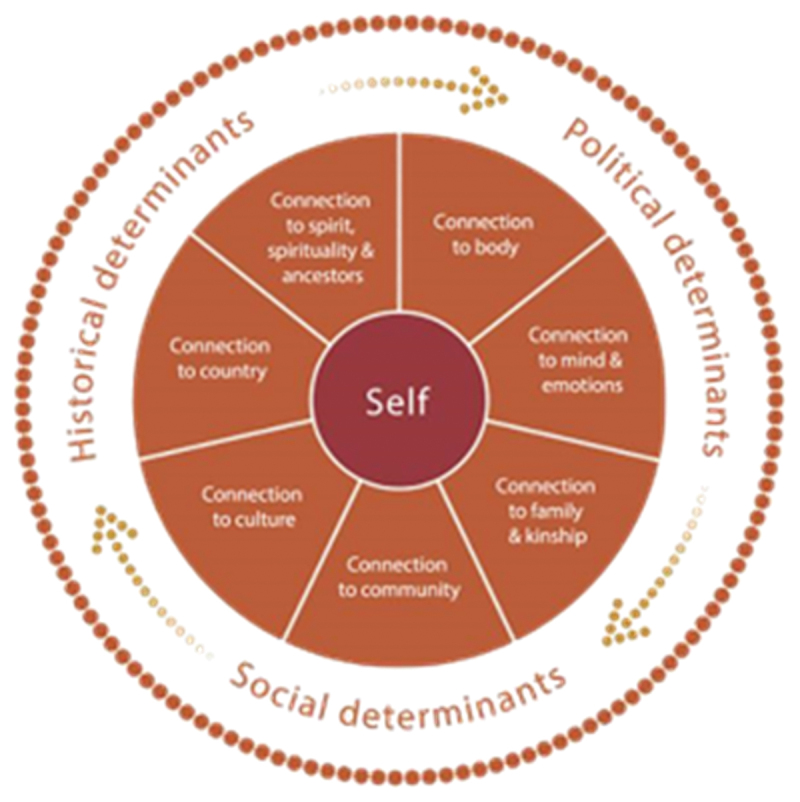
Social and emotional wellbeing model (Gee et al., 2014).

Aboriginal and Torres Strait Islander Peoples’ health is collective and holistic. Nurtured health at the individual and community level is a unique interplay of physical, social, emotional, spiritual, and ecological elements; conceptualised as “social and emotional wellbeing” (Dudgeon et al., 2014; Verbunt et al., 2021). This holistic perspective on health has enabled Aboriginal and Torres Strait Islander Peoples to sustain their wellbeing and persevere against colonial adversity (Dudgeon et al., 2014). Gee et al. (2014) social and emotional wellbeing model encapsulates the fundamental cultural and holistic needs and connections that serve as protective factors necessary for strength and healing; whereas disconnection from wellbeing needs perpetuate experiences of adverse outcomes (refer Figure 1) (Dudgeon et al., 2014; Verbunt et al., 2021). Fundamental to social and emotional wellbeing is connection to family and kinship which directly influences caregiving and child-rearing in Aboriginal and Torres Strait Islander communities (Brinckley et al., 2022; Gee et al., 2014). Together, these promote a sharing of knowledges, responsibilities, and duties of care between individuals and their communities. Aboriginal females have a core role in caring and nurturing children’s wellbeing; a concept referred to as multiple mothering (Ryan, 2011). In addition to biological mothers, grandmothers, aunties, sisters, and cousins have a shared and collective responsibility in educating, providing, and caring for children (Brinckley et al., 2022; Queensland Government, 2024; Ryan, 2011). Current evidence suggests Aboriginal mothers who adopt predominantly Western child-rearing practices due to growing up outside of culture can experience a compromise in their cultural identity and social and emotional wellbeing (Bailey & Clark, 2024).

### Conceptualising cross-cultural and western resilience

Psychological theory and practices are historically embedded within Eurocentric approaches; as such, these are not always culturally safe and informed when working with culturally and linguistically diverse populations (Azuma, 2007).

Whilst there is limited existing evidence to support a consistent definition of resilience from an Aboriginal perspective there are cross-cultural resilience understandings. Ungar’s (2008) cross-cultural resilience theory suggested that Indigenous peoples experience of ongoing colonial violence and adversity requires them to employ a myriad of holistic factors to foster resilience. Resilience is enriched through connection to cultural identity, cultural practices, drawing upon social connections, being on traditional lands, overcoming and healing intergenerational trauma, receiving an education, and connecting with emotions and cognitions (Goddard-Durant et al., 2023; Smallwood et al., 2024; Ungar, 2008; Wexler et al., 2014; Yadeun-Antuñano & Vieira, 2019). Resilience can also be nurtured through connection to a strong self-identity and flourishing mental health, all of which may be considered cultural strengths (Gartland et al., 2024).

Comparatively, Western theories suggest that resilience is an inherent, positive adaptation and behavioural technique employed at the individual level to achieve higher-level coping to overcome adversity (S. Hannon et al., 2023; Richards-Karamarkovich & Umamaheswar, 2024; Zlotnick & Manor-Lavon, 2023). Individuals determined to possess higher levels of cognitive functioning, optimism, and self-esteem nurture the ability to adaptively overcome stressful life events (Ledesma, 2014). Additionally, growing evidence suggests that resilience is contextual and dependent on social and environmental influences (Wilson et al., 2017; Zlotnick & Manor-Lavon, 2023). Resiliency may be fostered through connection to social networks, debriefing on distress, and self-care; all serving to alleviate stressors and nurture wellbeing (Aguillard et al., 2022; Bernardes et al., 2020; Bristow et al., 2022; Wilson et al., 2017; Zlotnick & Manor-Lavon, 2023). Subsequently, resilience may be formed, attained, or maintained at any point in life, dependent on necessity and context.

### Contextualising motherhood

Across cultures, motherhood is considered a pivotal and unique role, often one that encompasses enjoyable and positive experiences requiring her to make sufficient adjustments and become readily equipped with new knowledge and skills to provide infant care (Brinckley et al., 2022; Javadifar et al., 2016). However, such experiences can also perpetuate psychological distress and adjustment difficulties (Javadifar et al., 2016). The literature stipulates that a mother’s capacity to adjust within the maternal role is influenced by her beliefs, outlooks, intrapersonal experiences, culture, and own adverse experiences (Chamberlain et al., 2019; Javadifar et al., 2016). Bailey and Clark (2024) depicted the importance of Aboriginal mothers experiencing nurtured social and emotional wellbeing to foster their “parenting template”. Thus, Aboriginal mothers with positive experiences and practice methods in their role as caregivers are likely to pass this knowledge/template onto their child/ren to draw upon, in turn promoting mother and child wellbeing (Bailey & Clark, 2024). Subsequently, resiliency is a foundational component to flourishing in the maternal role, informed by unique experiences and circumstances (Bailey & Clark, 2024; Chamberlain et al., 2019; S. Hannon et al., 2023; Javadifar et al., 2016).

Nurtured wellbeing within motherhood is also achieved through a myriad of practices adopted as a source of empowerment and strength by mothers to inform strength.^2^ Growing social networks among mothers with shared experiences fostered resilience by providing an opportunity to de-brief difficulties and seek out guidance and advice (Bristow et al., 2022; Hinton & Earnest, 2010), and provides emotional support pivotal to flourishing and overcoming psychological distress (D’Amore et al., 2023; Haight et al., 2009; S. E. Hannon et al., 2022; Sanayeh et al., 2021). Empowerment may be formed through education, with mothers building the capacity to make informed decisions for their children’s care (Bristow et al., 2022; Goddard-Durant et al., 2023; Zlotnick & Manor-Lavon, 2023). Additionally, receiving education (training, higher education) and financial independence to provide for their child (Goddard-Durant et al., 2023; Hinton & Earnest, 2010). Literature that has identified trait-based processes for fostering resilience in mothers, such as optimism, indicateimproved skills in stress management and adaptive problem-solving. Such skills enable mothers to possess positive outlooks and foster positive wellbeing (Bristow et al., 2022; Sanayeh et al., 2021). Research into Aboriginal motherhood resilience and practices is scarce. However, research by Ussher et al. (2016) highlighted that Aboriginal mothers who sought to heal their trauma and manage stressors had greater coping capacity. In exploring Aboriginal mothers’ ways of knowing, being and doing post-partum, this research will assist in informing practitioners and services of how to support and nurture resilience in a culturally safe capacity, ensuring Aboriginal mothers have the best opportunity to flourish within the early years of their maternal role.

## Research questions

The study explores the perspectives of resilience through the following:

1. In what ways do Aboriginal mothers conceptualise staying strong and nurturing their strengths based on their experiences?
2. What needs to be further understood about Aboriginal mothers’ strength so that systems and services can be influenced and/or developed appropriately to provide sufficient support?

## Method

### Study context

Our research extends upon a South Australia Health and Medical Research Institute’s (SAHMRI) Women and Kid’s Theme mixed method study: “Corka Bubs, deadly mums, and strong families: connecting pregnant women with support for stress, yarndi or alcohol”.^3^ Corka Bubs provided a support package to Aboriginal mothers across two maternal birthing hospitals on Kaurna Yerta.^4^ The support package assisted with alleviating stressors and consisted of access to an anonymous Grog Survey App,^5^ a culturally informed counsellor, drug and alcohol worker, and legal practitioner. The Ngangkita Ngartu^6^ (Aboriginal Family Birthing Program [AFBP]) at the Women’s and Children’s Hospital (WCH) (a major birthing hospital) site employed a purposive sampling technique to recruit 40 pregnant mothers and four support persons. This sampling methodology offered unique insights and knowledge of a specific population group, enabling in-depth exploration (Palinkas et al., 2015). Corka Bubs sought to generate a new and culturally informed approach for community-based antenatal practices. It is hypothesised that findings from this study would generate preliminary evidence to improve post-natal health and wellbeing outcomes for Aboriginal mothers. The current project seeks to connect with mothers previously enrolled in Corka Bubs at one of the study sites to explore how mothers nurture post-natal strength and stay strong. The research team for the extended project comprised three Aboriginal women and one non-Indigenous ally.^7^ Two other Aboriginal women involved in the original Corka Bubs study have added to this project and article.

### Ethics, consultations and recruitment

Human Research Ethics Committee clearance was approved by the Aboriginal Health Research Ethics Council on the 13th of May 2024 (#04–24–1121); Women’s and Children’s Hospital (WCH) Human Research Ethics Committee (HREC) on the 29th of May 2024 (#2021/HRE00417); WCH Site-Specific Assessment (SSA) ethics on the 6th of June 2024 (#2022/SSA00155); and the University of South Australia HREC E1 on the 25th of June 2024 (206015).

Consultation with team leaders at Ngangkita Ngartu AFBP regarding the best approach to recruit mothers indicated that mothers may not readily have access to electronic devices and experience residential mobility. The first point of contact occurred via midwives visiting mothers in the birthing ward and notifying them of the extended study; participants were provided with an information pack comprising a cover letter, information sheet, and consent form. The second contact occurred through texts sent to 28 participants with an invitation to partake in the study. Nine individuals expressed interest and were contacted by the researcher to clarify questions, obtain verbal and written confirmation of informed consent, and schedule a yarn. Participants were screened to ensure they met the inclusion and exclusion criteria (refer Table 1). Telephone yarns then occurred for approximately 30–90 minutes to explore resilience in motherhood. The research was time-limited, intensive, and focused on collecting in-depth knowledge and stories from each participant.

**Table 1.** Inclusion and exclusion criteria for participants.

Inclusion	Exclusion
Aboriginal mothers previously engaged with Corka Bubs research.	Aboriginal mothers not participants of the original study.
Over the age of 18.	Those adolescent mothers under the age of 18 previously engaged in Corka Bubs.
A sufficient understanding and comprehension of English.	Mothers who do not speak or have extreme difficulties with comprehension of English language.
	Circumstances (such as mental health) that might interfere with the participant’s ability to provide informed consent (as determined by hospital staff, in particular an Aboriginal health worker or Aboriginal Maternal Infant Care worker based at the clinic)

### Participants

Of the nine responses received, five interviews were completed. Mothers interviewed varied in age, number of children, and Aboriginal language groups or Mobs^8^ (refer Table 2). The project experienced recruitment challenges due to the short enlistment timeframe coupled with complex demands of mothers, with several experiencing wellbeing issues and child illness requiring hospitalisation. Post yarn, participants received a welfare check-in and a gift of appreciation for their generous input. To uphold confidentiality, pseudonyms in the form of numbers in the Kaurna language were respectfully employed (i.e., participant 1 = Kuma and so forth to number 5[Mila]). As the research was conducted on Kaurna country, adopting Kaurna language aided in the Indigenous and decolonising methodological framework. The interviews produced rich and meaningful data, and the information gained was adequate for achieving saturation and triangulation with no new themes emerging within the data (Braun & Clarke, 2021; Hennink et al., 2017).

**Table 2.** Participant information.

Participant	Age (mother)	Age (child/ren)	Mob
Kuma	25	11 months	Noonuccal & Goenpul
Purlaityi	21	3 months	Pitjantjatjara, Yankunytjatjara, & Arrernte
Marnkutyi	29	3.5 months	Ngarrindjeri
Yarapurla	25	5 years, 4 years, 2 years, and 4 months	Ngarrindjeri
Mila	32	14 years, 13 years, 11 years, 9 years, 7 years, 5 years, 7 months	Kokatha

### Data collection and data analysis

The qualitative research was grounded within an Indigenous methodology that was strength based and promoted cultural safety by incorporating Indigenous ways of knowing, being, and doing (Martin-Mirraboopa, 2003). Thus, avoiding harm perpetuated by historically unsafe, inappropriate and ill-informed Eurocentric methodologies (Keikelame & Swartz, 2019). Exploratory analysis was fostered through a yarning-style approach to promote unique storytelling, in-depth learning, and relational understandings; whilst minimising power dynamics between participant and researcher (Bessarab & Ng’andu, 2010; Geia et al., 2013; Leeson et al., 2016; Walker et al., 2014). Behrendt (2023) advocates that an Indigenist methodological standpoint comes with an understanding that Indigenous people are “shaped by culture, cultural values and experiences within societies and institutions” (p. 176). As Indigenous researchers subjectivity is important to understand the realities of the research knowledge holders.

Interviews were recorded, transcribed, and uploaded into NVivo 12. Braun and Clarke’s (2006) six-step process to thematic analysis was applied to examine the data, offering a rich and unique exploration into resilience experiences. The data analytic strategy followed an inductive approach, coding at both semantic and latent levels, whereby themes were informed by underlying assumptions, ideas and knowledges and directly linked to participant responses (Braun & Clarke, 2006). Where possible, names of themes were derived from the participants’ words to privilege Aboriginal mothers’ knowledges and voices.

Methodological integrity was upheld through analysis and decision-making processes being debriefed and cross-checked by senior, experienced researchers during weekly supervisory meetings. Further, the research adhered to SAHMRI’s nine principles of the South Australian Aboriginal Health Research Accord (Morey et al., 2023), to uphold cultural safety and ethical practice (refer Table 3). The Aboriginal and Torres Strait Islander Quality Appraisal Tool ensured all aspects of the research remain in the community’s best interest and promote cultural safety (refer Table 4) (Harfield et al., 2020).

**Table 3.** South Australian Aboriginal Health Research Accord.

Initiative	Description
1. Priorities	Research to be informed by Aboriginal community priorities to promote acceptability, relevance, and accountability.
2. Involvement	Involvement between Aboriginal community and organisations across aspects of the research.
3. Partnership	Establishing connection and trust to work cross culturally.
4. Respect	Researchers uphold respect for Aboriginal ways of being, knowing, and doing.
5. Communication	Relevant to culture and receptiveness of community.
6. Reciprocity	Research provides benefit to the community aligned with their priorities.
7. Ownership	Respect for ownership of data and intellectual property of Aboriginal community.
8. Control	Respectful and culturally relevant ways of research management.
9. Knowledge Translation and Exchange	Sharing of evidence integrated into all approaches of research for maximum impact and benefit.

*Note.* Table adapted from Research ACCORDing to whom? Developing a South Australian Aboriginal and Torres Strait Islander Health Research Accord. (https://doi.org/10.1016/j.fnhli.2023.100003).

**Table 4.** Aboriginal and Torres Strait Islander quality appraisal tool.

Question	Researcher Response
1. Did the research respond to a need or priority determined by the community?	Yes, the research provides further contributions in supporting Aboriginal mothers right to self-determination, ultimately working towards closing the gap between Aboriginal and non-Indigenous peri and postnatal health outcomes.
2. Was community consultation and engagement appropriately inclusive?	Yes, multi-layer community engagement and consultation were sought out through the wider Aboriginal maternal community and in combination with Aboriginal researchers from SAHMRI.
3. Did the research have Aboriginal and Torres Strait Islander leadership?	Yes, Aboriginal leadership was prioritised in both the original Corka Bubs study, and the extended study. The current study ensured that the supervisory team comprised two Aboriginal researchers who were able to guide the Aboriginal student researcher surrounding best practices and decolonising research approaches.
4. Did the research have Aboriginal and Torres Strait Islander governance?	No, the extended study did not have Aboriginal Governance due to the time constraints of the Master’s program. Before publication, Aboriginal Governance will be sought to ensure reflections of the community are culturally informed and accurate.
5. Were local community protocols respected and followed?	Yes, protocols were consistently respected with the student researcher upholding cultural safety practices throughout the entirety of the study.
6. Did the research negotiate agreements in regard to the rights of access to Aboriginal and Torres Strait Islander peoples’ existing intellectual and cultural property?	Yes, several ethical reviews and amendments were undertaken to ensure that the intellectual and cultural property of the community was embedded within the research.
7. Did the researchers negotiate agreements to protect Aboriginal and Torres Strait Islander peoples’ ownership of intellectual and cultural property created through the research?	Yes, consistent with a decolonising and Indigenous methodology framework, Aboriginal mothers remained owners of their data.
8. Did Aboriginal and Torres Strait Islander peoples and communities have control over the collection and management of the research materials?	Yes, the current research privileged participants’ management over research materials; i.e., review of transcripts with access made available upon request, and the right to withdrawal post data finalisation.
9. Was the research guided by an Indigenous research paradigm?	Yes, the extended study’s research and analysis was led by an Aboriginal woman in conjunction with support from her supervisory panel comprised of two experienced Aboriginal researchers and clinicians.
10. Does the research take a strengths-based approach, acknowledging and moving beyond the practices that have harmed Aboriginal and Torres Strait peoples in the past?	Yes, the research privileged a strength-based approach and ensured that those successes and positive experiences of resilience in motherhood were emphasised.
11. Did the researchers plan and translate the findings into sustainable changes in policy and/or practice?	Yes, the intention throughout the study was to ultimately translate findings to policy and practice; with findings to be disseminated back into community. The current research intends to be published and findings shared.
12. Did the research benefit the participants and Aboriginal and Torres Strait Islander individuals?	Yes, participants in the study benefited from sharing their stories as it allowed them to reflect on their strengths throughout motherhood, foster self-empowerment, and experience positive emotions. Moreover, mothers were provided with $50 vouchers as reimbursement for their time and thanks for sharing their stories.
13. Did the research demonstrate the capacity strengthening for Aboriginal and Torres Strait Islander individuals?	Yes, the research focussed on reflecting upon mothers’ strengths.
14. Did everyone involved in the research have opportunities to learn from each other?	Yes, the study provided several instances whereby individuals were able to learn from each other. The researchers had an opportunity to learn from mothers’ stories of resiliency and were able to learn from each other professionally by sharing perspectives and knowledge in supervision.

*Note.* Table adapted from Assessing the quality of health research from an Indigenous perspective: the Aboriginal and Torres Strait Islander quality appraisal tool. (https://doi.org/10.1186/s12874-020-00959-3).

## Results

**Figure 2.**
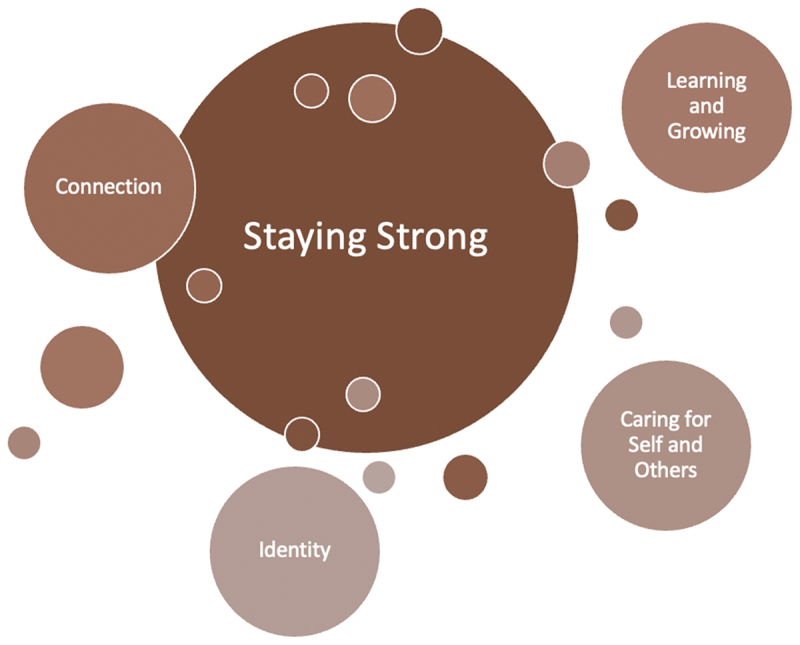
Conceptual map of core findings.

The analysis revealed that Aboriginal mothers’ resilience is grounded in unique ways involving strength-based approaches and holistic understandings. The four themes are reflected in Figure 2’s conceptual map; at its centre, staying strong is informed by diverse elements that collectively nurture resilience. Mothers described the importance of upholding connection to family, kinship, and community. Inclusive of this connection was fostering strength and enabling proactive care for their child. Moreover, being receptive to learning and growing skills and techniques was important to build resilience. Mothers strived to be a positive role model for their children, an undertaking which cultivated strength and purpose. Mothers who practiced self-care alluded to possessing a greater capacity to uphold resilience and provide in-tune care to their children. Lastly, strength in identity was identified as a core component of motherhood resilience.

### Connection

Protective cultural factors inclusive of connection with family, community, and kin were widely referenced as a means of resilience in motherhood. Mothers spoke of the importance of connecting with female family members, particularly mothers, aunties, and sisters to share child-rearing responsibilities, in turn alleviating stress but also providing opportunities to stay connected with family. This mother articulated the collective child-rearing experiences within Aboriginal culture.

**Purlaityi:**
Because it’s my first baby, and for a first-time mum, you know, Aboriginals, when they have their first babies, mums, aunties, and sisters they all come down and support.

Important to mothers was being supported unconditionally by family and reliance on connection with these individuals to create a safe and loving environment for children.

**Yarapurla**: So, definitely having [partner] and such a support from him and his family is absolutely lovely, they’ve taken on me and my kids like we have been there for years, so it’s definitely comforting having that kind of family unit that have just gone “no worries”, like these are our grandkids too now and accepting everyone with open arms.

Participants also described how connection to their children facilitated a sense of purpose and belonging. Specifically, being attuned and connecting with them through being present enabled them to remain resilient through the hardships of motherhood. One mother described how the bond between herself and children provided self-regulation.

**Mila**: I’m so blessed, I guess, with the kids. Like when I see them smiling, or I come home to them after the worst day ever to my babies, it just relaxes all the nerves in my body.

The notion of being able to depend on family networks to support your own needs and thus, facilitate meaningful connection with babies was alluded to. Particularly, family who stepped in as caregivers allowed mothers to take time away from their role and rest and recuperate. This allowed for greater meaningful connection with their children upon returning to their caregiver role.

**Purlaityi**: For me, we get to spend a lot of time together, reading books with him and doing playtime with him.

Connection with family also served as a protective factor of strength and identity. Mothers privileged connection with familial networks to provide a sense of purpose.

As **Marnkutyi** described: To keep those relationships strong, yeah, you know, just makes you feel more like you belong somewhere.

Evidently, mothers valued connecting with familial networks to remain supported, facilitate quality time, and receive guidance, all of which deepened their ability to remain resilient in motherhood.

### Learning and growing

Mothers detailed the value of learning from others to harness resilience, and further, serving as a role model to children provided a purpose for staying strong. Emphasis was given to the role of family and how they serve as educators for learning and evolving in the role of motherhood. Being open to learning and using these skills in motherhood was important to remaining resilient. As this mother describes, it was paramount to learn in a way that was adaptive and healthy, and not punitive.

**Purlaityi**: I learned from my family, it’s kind of like they’re my teachers … sometimes when I’m doing something wrong, they don’t growl, they just tell me you don’t do it that way. It’s kinda like a puzzle.

Participants described experiencing self-doubt in their capacity to meet the caregiver’s role. Therefore, the guidance and the sharing of wisdom from family, served as a protective cultural factor to overcome doubt. This highlighted the importance of connection to family and the role of generational knowledge sharing and underpinned motherhood resilience.

**Mila**: I didn’t think I could do it, I was only 16 when I moved out, for the first year too … I was scared. So, I’m happy I had my mum around me as a role model for me and the baby. She would teach me everything I needed to know.

Mothers valued modelling positive and malleable means of coping to their children. Participants highlighted being a safe and responsive role model to their children, being emotionally connected, promoting further generational resilience, and learning. The notion of leading by example was important to fostering self-resilience.

**Yarapurla**: Um, I think just knowing that the kids have someone decent to look up to, like having that kind of strong woman presence … I definitely want to be emotionally available for my kids, I feel like that is what I want my biggest strength to be.

Moreover, mothers acknowledged that thriving can be challenging, however, their children provided purpose to persevere. This mother shared the importance of being a functional role model to her children and nurturing her resilience:

**Mila**: It gives me purpose to go do the right thing and to show my kids there is good in the world and you can do the right thing and achieve things.

Emphasis was placed on cultural learning and by surrounding children in culture, this nurtured strength, Aboriginal heritage, identity, and purpose.

**Kuma**: I’m teaching him everything I can, I mean he’s not that old yet … so just taking him to cultural events and having him around family and Mob.

### Caring for self and others

The stories shared by Aboriginal mothers revealed self-care as fundamental to their resilience. A range of mechanisms were shared including: accessing services, engaging in activities both socially and individually, meeting basic care needs, and routine engagement. Accessing mental health services was emphasised by mothers as it ensured they were able to effectively manage their wellbeing and had the confidence to continue with counselling post-pregnancy.

**Marnkutyi**: I kind of struggle with my mental health … so in my pregnancy, I definitely utilised the counselling services that were in the hospital, that just kind of opened me up to being able to get more help with therapy … so I’ve been going to therapy every fortnight since she was born.

Participants additionally shared that being connected and linked with wellbeing services was important for their self-care journey. As detailed by this mother, access to services enabled her to receive professional support in addressing cognitive distortions.

**Mila**: I was able to stay strong, I love Corka Bubs because I actually talked to counselling, and they drove me in the right direction because I was all thinking negative, negative, negative.

Mothers demonstrated insight regarding the importance of mental health intervention. Specifically, participants acknowledged that by looking after themselves, they had a greater capacity to effectively care for their children.

**Yarapurla**: I’m now slowly starting to go, alright, no, at the end of the day the kids need a happy, healthy mother, so I reached out to get some kind of mental health help and trying to find a domestic violence specialised psychologist.

A proportion of mothers in this study cared for children with complex developmental needs. It was a priority for participants to access services that could aid and support their children’s needs. Ensuring their children were cared for and needs were met facilitated maternal strength.

**Mila**: I didn’t even know there was an extra payment there that you can claim if you’ve got kids with disabilities … . So, I actually got that this year, and I put that away because I’m building a house … the housing trust is taking so long to get us a house suitable for the needs of the children, just ridiculous.

Mothers detailed the importance of engaging in activities, whether this be for themselves or with others. Having time away from caring for their children and being able to engage in enjoyable activities provided energy and motivation for when caring for children was important. This mother described being able to depend on family whilst she connected with her partner enabled resilience.

**Kuma**: Getting mum to watch the baby whilst me and my partner go out … sometimes we go for dinner or have a drink or two.

Participants described the enjoyment of being by themselves to help stay strong through motherhood.

**Marnkutyi**: I don’t really get much alone time, so anything doing by myself is pretty nice.

Mothers emphasised that caring for themselves was also important to provide adequate care for their babies.

**Kuma**: I think taking time out for myself really helps so I don’t get overwhelmed with looking after baby.

Ensuring that mothers also fulfilled their basic care needs was a component of remaining resilient. Participants spoke of simple tasks such as bathing, sleeping, and nutrition as important self-care, in turn meeting basic care needs fosters strength.

**Marnkutyi**: I take a bath or something like that.

**Mila**: Just have a long bath I guess and get my hair cut.

**Purlaityi**: Soon as my mum got here, I was getting good sleep and a decent feed, started back to my routine, got back to where I was.

Participants emphasised having structure and routine allowed them to meet their own needs whilst balancing the role of motherhood. Particularly, assistance from support networks to create a functional routine assisted in remaining resilient.

**Marnkutyi**: Just using the strategies that I’ve learnt through counselling. So, trying to find like a routine and um … just different ways to stay positive

Guidance from Elders and generational learning encouraged mothers to use adaptive coping mechanisms like routines to have optimal functioning.

**Purlaityi**: I started on a little routine … my aunty come down and tell me to do a routine, like what time to feed baby, when he’s going to wake up, then when I should get up and have a shower … yeah so my aunties that came down really supported me with my routine, I told them that’s what’s been keeping me going, that routine.

Moreover, participants briefly mentioned how being away from home, specifically, within nature, was also a practical strategy for managing wellbeing. Nature was enjoyed through connection to family.

**Marnkutyi**: Go for walks in nature, that’s kind of the whole family thing.

**Purlaityi**: My big sisters, they would take me out for a walk and encourage me to do things … have a confident mind.

### Identity

The notion of being strong within yourself stemmed from a myriad of intrapersonal qualities harnessed by mothers. Participants spoke of their identity allowing them to persevere through the challenges of motherhood. Specifically, perseverance stemmed from mothers being strong-willed, determined, collectivist, confident, and optimistic. Mothers emphasised having a strong-willed attitude to facilitate perseverance and “survive” motherhood. The quote below also indicates how these intrapersonal qualities serve to promote altruism.

**Marnkutyi**: I’ve definitely had to become a lot more resilient. Like you just kinda have to get up and do it, can’t sit in bed all day, you have someone to take care of. It’s taught me that taking care of someone other than myself is pretty important.

Moreover, mothers explicitly stated that it was an inherent action and skill they possess to persevere and overcome challenges. Specifically, an automatic and unconscious ability to be able to make it through the day.

**Marnkutyi**: Just mostly trying to survive every day … yeah, you just get on with it and do what you can do.

Additionally, this participant shares the concept of perseverance, proving yourself wrong, and moving beyond your doubts.

**Kuma**: I just know I have to do it, so I do it … that I can actually do it, I didn’t think I could.

Qualities of optimism, confidence, and a “never giving up” attitude were further articulated by mothers as core components of fostering resilience. Mothers implied that adopting positive outlooks, not only allowed them to be strong within themselves but was critical to surround their children with positive attitudes.

**Marnkutyi:**
Just trying to stay positive and not get into like sort of a negative mindset … stay positive for the baby.

A sense of optimism and hope and instilling this within your children to be resilient was further described. Mothers shared that it was important to have hope to adapt and overcome.

**Yarapurla**: Definitely showing them that just because things knock you back, don’t give up, there’s something out there for you.

Ultimately, intrapersonal qualities that mothers possess may promote their capacity to put others first and provide care for others above their own needs. The combined traits of being strong-willed and confident, fostered thriving and succeeding with confidence by ensuring their babies and children were cared for.

**Mila**: Yeah, I feel good in myself for not giving up. Like, thinking that I was hopeless or something like that. But never giving out is my goal. I’ll just prove to myself I can do it, if I put my mind to it, I can save the money, and I will save the money because it’s for my children’s wellbeing.

## Discussion

The stories shared regarding Aboriginal mothers’ strengths and ways of staying strong broadened perspectives of resilience to encompass strength-based and holistic understanding necessary to future development of culturally informed supports. The unique stories related to the mother’s holistic Aboriginal cultural backgrounds and knowledges and revealed a crossover with Western resilience modalities. This suggests that they are operating within two cultural foundations. The insights by Aboriginal mothers were consistent with those of Indigenous resilience theories and emphasised connectedness to culture and communities, and generational learning (Goddard-Durant et al., 2023; Smallwood et al., 2024; Ungar, 2008; Wexler et al., 2014; Yadeun-Antuñano & Vieira, 2019). Ultimately, post-natal resilience is a core component of adaptive functioning for Aboriginal women and cannot be overlooked when seeking to support their post-natal wellbeing needs.

The mothers implied that Aboriginal post-natal resilience is underpinned by protective factors that align with the holistic nature of social and emotional wellbeing. Fundamental to resilience was privileging connection to culture, community, family & kinship, and mind, body, and emotions (Dudgeon et al., 2014; Gee et al., 2014; Verbunt et al., 2021). Such holistic understandings emphasised the importance of remaining connected to a myriad of protective factors to foster learning, connection, safety, self-care, and wellbeing (Dudgeon et al., 2014; Gee et al., 2014). Our study corroborates recent findings of Gartland et al. (2024) in that motherhood resilience (as in Aboriginal children) can be nurtured through their basic needs being met, family connection, having confidence and pride. Combined, these findings suggest there are multiple factors in nurturing resilience for Aboriginal communities that are relevant across the life span.

Connections to all social and emotional wellbeing domains is important for Aboriginal people to nurture their identities and activate their protective factors. However, during our yarns, there was little mention of connection to spirituality/ancestry in nurturing mothers’ strength (Gee et al., 2014). It may therefore be understood that certain domains of social and emotional wellbeing are more relevant than others dependent on circumstances and experiences across certain life phases such as in post-natal periods. As many mothers indicated, they were operating in two worlds, for survival and as a protective factor. Nevertheless, if elements of a Western knowledge system are dominant, then Aboriginal people may not experience nurtured social and emotional wellbeing and may seek to strengthen connections to Aboriginal culture. The social and emotional wellbeing framework emphasises connectivity (Gee et al., 2014). In the study stronger connections to culture, community, family, and body over other wellbeing elements may dominate especially if more holistic or collective family support is needed to care for the baby. A tailored understanding of social and emotional wellbeing needs is critical to supporting Aboriginal post-natal resilience; as such, clinicians should be encouraged to work flexibly, holistically and draw upon pre-existing models to nurture strength.

Cultural ways of knowing, being and doing were identified as a critical enabler for resiliency. Mothers emphasised the central role of connecting with significant Aboriginal women to nurture their resilience and support them with children’s care needs, informing them on ways of caring, and alleviating stressors by sharing the child-rearing experience. This finding is consistent with information about the important role of multiple mothering and collective child-rearing in Aboriginal communities (Bailey & Clark, 2024; Brinckley et al., 2022; Ryan, 2011). Evidence suggests that positive experiences form “parenting templates”, which are then passed from mothers onto their children’s, this cultural practice leads to improved wellbeing outcomes (Bailey & Clark, 2024; Brinckley et al., 2022). The mothers of this study embodied this cultural practice and embraced collective child-rearing experience to ensure that not only their wellbeing was nurtured, but also their children; this was paramount to reducing stressors and remaining strong. Furthermore, Brinckley et al. (2022) detailed the core role of Aboriginal female members as important figures in educating Aboriginal mothers via shared Aboriginal knowledges. Ultimately, shared generational knowledge for Aboriginal mothers’ experience of resilience must be informed by embedding cultural child-rearing knowledges.

Distinctions can be drawn between Western models of resilience and the current study. It may be tempting to assume that non-Indigenous and Aboriginal mothers remain resilient through connection to support networks; however, it is vital to acknowledge the differences underpinning their practice of doing so. Western resilience studies highlight that mothers depend on social networks for emotional support and to debrief stressors (Bristow et al., 2022; D’Amore et al., 2023; Haight et al., 2009; S. E. Hannon et al., 2022). Contrastingly, Aboriginal mothers found strength in connecting with community and kinship structures, for learning and sharing of child-rearing responsibilities, which is a long held cultural practice. This distinction is imperative, highlighting Aboriginal women do not solely connect with their networks for yarning, but rather, utilise them to foster self-growth for adaptive functioning. Systems supporting Aboriginal maternal wellbeing are grounded in a deficit framework, hindering engagement and wellbeing outcomes for community. Our findings indicate the importance of empowering Aboriginal women with their strength through systems that collectively nurture cultural learning, and knowledge sharing.

Eurocentric and individualistic perspectives of motherhood resilience reflect traits as an important component of resilience; this was shared by Aboriginal mothers. Optimism and maintaining a positive outlook foster capacity to overcome adverse events and achieve positive functioning (Bristow et al., 2022; Sanayeh et al., 2021). Aboriginal mothers reflected maintaining a strength-based focus and not being weighed down by negative emotions and experiences to adaptively persevere. This finding aligns with pre-existing research evidence suggesting intrapersonal traits have a core component in resilience across all cultures (American Psychiatric Association, 2020; Sanayeh et al., 2021). The importance of prioritising strength-based traits, rather than remaining passive, may be understood within the context of colonisation. It is well documented that Aboriginal mothers experience a disproportionate rate of adversity in their maternal role due to ongoing colonial violence (Simpson et al., 2020; Sivertsen et al., 2022; Ussher et al., 2016). Impacts of intergenerational trauma may therefore require Aboriginal mothers’ adaptability and fostering of these traits for self-preservation and to manoeuvre systems based on Western values and practices. Mothers in this study reflected “I just have to do it and so I do it” and “trying to survive”, this may imply that mothers must remain strong-willed, determined, and optimistic as a means of survival in motherhood due to systemic experiences of continuous colonisation. Subsequently, it must be acknowledged that whilst there are shared personality and behavioural components of motherhood resilience between Aboriginal understandings and Western trait-based literature, their manifestation differs based on colonial context. For example,determination and optimism in Western literature are harnessed at an individual level for mothers’ self-preservation (Bristow et al., 2022; Sanayeh et al., 2021). In contrast our study revealed mothers utilised determination and optimism for positive role modelling for their children, allowing them to learn (by doing) more adaptive means of overcoming adversity. This novel distinction suggests Aboriginal mothers employ collectivist values to nurture resilience, with the value of growing healthy adaptive functioning for future generations. Our findings suggest that Aboriginal mothers' experience of resilience more closely aligns with global Indigenous theories of resiliency and whilst there are similarities within Western resilience, the context differs in respect to cultural values and colonisation. Paramount is the connection to kinship, family, and community as protective factors of motherhood resilience and disconnection to these factors may perpetuate adverse outcomes (Bailey & Clark, 2024; Gee et al., 2014; Usher et al., 2021). These findings emphasise collectivism, illustrating that Aboriginal mothers find strength in nurturing resilient children and generational learning. Therefore, there is a critical need for policies and services to deliver post-natal supports that are holistically and collectively focused.

Aboriginal mothers reflected self-care as an essential component of staying strong. Research has established the link between the usage of self-care and its reduction in burnout (Wilson et al., 2017; Zlotnick & Manor-Lavon, 2023) and to overcome experiences of chronic stress and trauma (APA, 2020; Bristow et al., 2022; Sanayeh et al., 2021). Given experiences of colonial violence and consequently ongoing intergenerational trauma, such as fear of child protection, potential removals, and interactions with the criminal justice system (Clark et al., 2021); nurturing resilience in Aboriginal mothers is vital to flourishing. For Aboriginal mothers, self-care may be understood through a social and emotional wellbeing lens (Gee et al., 2014); connection to body helps protect and strengthen their mind and emotions for nurtured resilience. Our findings support pre-existing evidence and the notion of self-care as an adaptive protective factor of motherhood resiliency. Our research offers important implications for service delivery and system reform. Aboriginal communities access mental health services at a rate three times more than non-Indigenous Australians (Bailey & Clark, 2024); despite these rates, wellbeing services have proven to be culturally unsafe and ill-informed (Dawson et al., 2023; Vicary & Westerman, 2004). Our mothers identified access to services as a fundamental component of resilience. Culturally safe wellbeing services were accessed as part of Corka Bubs, with an opportunity for limited follow-up post pregnancy. Such safe services that are informed by Aboriginal ways of knowing, being and doing need to be offered post pregnancy on an ongoing basis. This study supports the call to action: wellbeing services must be accessible and informed using strength-based approaches encompassing cultural knowledge to facilitate self-determination and empowerment in Aboriginal mothers’ resilience journeys (Bailey & Clark, 2024; Dawson et al., 2023).

## Research strengths and limitations

Our project is a significant contribution to the literature and Aboriginal wellbeing more broadly. The project was led by a team of Aboriginal women, for Aboriginal mothers, providing critical implications to meet wellbeing needs of Aboriginal mothers in the peri- and post-natal period. In exploring a nuanced understanding of the practices surrounding resilience embodied by Aboriginal mothers this ultimately seeks to strengthen thriving for babies and mothers in their first 1000 days of life and beyond. The study’s Indigenous methodological approach upheld self-determination and privileged those practices and knowledges of Aboriginal Peoples, whilst remaining strength-based and promoting cultural safety. The authors acknowledge the small sample size and the limitations respective to reflecting a thorough scope of resilience perceptions. However, the sample was sufficient to achieve data saturation with corroborating stories and understandings. As with most qualitative data, the study’s findings are not generalisable to all Aboriginal mothers, especially as the participants comprised those living on Kaurna Yerta. Nevertheless, it provides in-depth stories about the needs and strengths of various Aboriginal mothers and is a great reflection of their stories. Whilst participants’ Mobs varied, it is important to understand Aboriginal cultural diversity, and even if there was a vast array of participants, no study would be culturally generalisable to the entirety of Australia’s Aboriginal population. Moreover, these participants were already engaged within an Aboriginal maternal service; and enrolled in the original Corka Bubs research project which enabled access to wellbeing services that were co-located at the AFBP. Understandably this limits diverse resilience perspectives from mothers who did not have access to Corka Bubs services. Future research would benefit from examining and comparing the experiences of resilience from a wider post-pregnancy maternal cohort, one encompassing diverse Mobs and ages. This would ultimately lead to a further in-depth understanding for clinicians to deliver tailored support services.

## Implications for policy and practice

This study offers insight into Aboriginal post-natal experiences of resilience, an understudied field of psychology. This exploration was critical, with findings serving to inform the supports, services, and interventions available to Aboriginal mothers, given the known adversities and systemic challenges these women endure. Our yarns with Aboriginal mothers offer a unique lens of resilience beyond the Eurocentric understanding. This provides an opportunity for system-level reform to embed knowledge and practices that are culturally informed into services provided to Aboriginal mothers’ post pregnancy. Importantly, Aboriginal mothers should be supported with the understanding and recognition that Aboriginal women are survivors and possess resilience, and that clinicians need to nurture and support post-natal resilience through a trauma informed lens and traditional ways of being and doing.

As an extension of the Corka Bubs project, important implications are gleaned for continued development of care packages for Aboriginal mothers. Providing safe psychoeducation that encompasses the principles of resilience revealed in this study: connection to kin and family, self-care and identity may facilitate self-empowerment for the development of resilience practices in motherhood and nurture social and emotional wellbeing. Using evidence informed understandings, we may increase engagement in pre- and post-natal services, which have historically been drastically low compared to non-Aboriginal mothers; this, in turn, may promote nurtured and flourishing communities in addressing psychological needs.

## Conclusion

Exploring Aboriginal mothers’ resilience promoted an informed understanding of how staying strong manifests for this population. Our aims were supported by uncovering diverse factors relevant to Aboriginal postnatal resilience and providing important implications for practice, policy development, and system reform. These insightful findings suggest protective factors of connection to mind and body (i.e., intrapersonal traits and self-care) and connection to family and kin to draw upon generational knowledge sharing and learning for support serve to nurture resilience. Findings from the study share resilience knowledges as depicted in Western literature; however, the contextual reasons underlying Aboriginal mothers’ resilience differ based on historical experiences, and collectivist and holistic understandings. Practitioners and service providers seeking to nurture resilience are encouraged to acknowledge Aboriginal mothers as experts in their experiences and privilege their traditional ways of being, knowing and doing.

## Data Availability

The data that support the findings of this study are available on request from the corresponding author, CS. The data are not publicly available due to their containing information that could compromise the privacy of research participants.

## Notes

1. “Aboriginal and Torres Strait Islander” will be referred to based on context and literature, whilst “Aboriginal” is used to describe the focused population relevant to the study and its findings.

2. Terminology of “strength” and “staying strong” will be utilised interchangeably with resilience to reflect strength-based perspectives of health and wellness for Aboriginal communities.

3. Hereafter referred to as “Corka Bubs”.

4. Kaurna Yerta is the language of the Kaurna people used to described their traditional land of the Adelaide region.

5. Grog Survey App was designed for Aboriginal community members to anonymously report their alcohol usage, with this data used to inform government alcohol support and intervention programs.

6. Ngangkita Ngartu’, is the Kaurna term referring to the Aboriginal Family Birthing Program.

7. Positionality of research team members can be found in the appendix.

8. “Mobs” is a term in Aboriginal cultures that distinguishes the Country, and or place, or language group an individual belongs to.
